# Real time phase encoded MR for assessment of acute variability of central pulse wave velocity

**DOI:** 10.1186/1532-429X-14-S1-W51

**Published:** 2012-02-01

**Authors:** Nicholas Gaddum, Tobias Schaeffter, Philipp B Beerbaum

**Affiliations:** 1 Imaging Sciences, King's College London, London, UK

## Background

The variability of pulse wave velocity (PWV) with changing arterial wall stress may reflect the degree of aortic stiffness independent of arterial pressure. We implemented a real-time MR flow technique so as to be able to assess acute changes in PWV due to affected arterial blood and/or intra thoracic pressure. The aim of this study was to assess the accuracy of rapid PWV assessment using the real time MR technique.

## Methods

We developed a novel real-time MR dual-slice protocol (RT-DS), with interleaved through-plane acquisitions of projected left to right velocity to rapidly measure central pulse wave velocity (PWV). An automated in-house post-processing tool measures mean velocity pulse wave transit time and aortic length, permitting PWV assessment from projected image data. In a pulsatile flow phantom, RT-DS-derived PWV was validated against pressure-wire-PWV (gold-standard) and in-plane 2D phase-contrast MRI (PC-MRI). In 17 healthy adult volunteer scans (mean age, 32 ± 7; mean arterial blood pressure 89 ± 7 mmHg), agreement of PWV by RT-DS was compared with in-plane PC-MRI.

## Results

Excellent correlation of assessed PWV between pressure wire (6.35 ± 0.18 m/s) and both RT-DS and PC-MRI scans were observed, (6.20 ± 0.61 m/s, P = 0.70, and 6.33 ± 0.70 m/s, P=0.96, respectively), in vitro. Volunteer velocity profiles with located wave ‘foot’ locations using both MR scanning protocols are shown in the figure below for comparison. In the volunteer cohort, the RT-DS and PC-MRI scans showed a correlation of 0.9, (r2=0.25, P > 0.04). RT-DS derived PWV was lower than standard PC-MRI (mean difference, -0.46 ± 0.78 m/s). Inter-scan variability of PWV by RT-DS was 0.41±0.32 m/s. Regional PWV between ascending/descending and descending/abdominal aorta lacked reproducibility.

**Figure 1:**
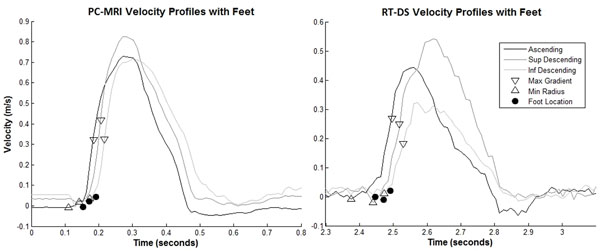
PC-MRI (left) and RT-DS (right) velocity profiles with located wave ‘feet’ from a volunteer scan

## Conclusions

Real-time dual-slice projection phase-contrast MRIS is accurate and reproducible to assess the aortic PWV. The method has potential to detect acute the variability of PWV in response to variation in arterial blood and/or intra thoracic pressure as a measure of arterial stiffness.

## Funding

Medical Research Council, UK.

